# The Limits to Estimating Population-Genetic Parameters with Temporal Data

**DOI:** 10.1093/gbe/evaa056

**Published:** 2020-03-29

**Authors:** Michael Lynch, Wei-Chin Ho

**Affiliations:** Biodesign Center for Mechanisms of Evolution, Arizona State University

**Keywords:** population genomics, effective population size, selection coefficient, fluctuating selection

## Abstract

The ability to obtain genome-wide sequences of very large numbers of individuals from natural populations raises questions about optimal sampling designs and the limits to extracting information on key population-genetic parameters from temporal-survey data. Methods are introduced for evaluating whether observed temporal fluctuations in allele frequencies are consistent with the hypothesis of random genetic drift, and expressions for the expected sampling variances for the relevant statistics are given in terms of sample sizes and numbers. Estimation methods and aspects of statistical reliability are also presented for the mean and temporal variance of selection coefficients. For nucleotide sites that pass the test of neutrality, the current effective population size can be estimated by a method of moments, and expressions for its sampling variance provide insight into the degree to which such methodology can yield meaningful results under alternative sampling schemes. Finally, some caveats are raised regarding the use of the temporal covariance of allele-frequency change to infer selection. Taken together, these results provide a statistical view of the limits to population-genetic inference in even the simplest case of a closed population.

## Introduction

Most evolutionary features ultimately depend on the magnitudes of a few key population-genetic parameters—rates of mutation, recombination, and migration, the strength and pattern of selection, and the stochastic noise caused by random genetic drift. As the magnitudes of these forces are typically quite small at the molecular level, most estimates derive from observations on standing patterns of variation in samples of multiple individuals acquired at a single time point, which are typically assumed to reflect cumulative, equilibrium effects. A diversity of methods relies on such strategies, including those focused on variation at genomic sites or on the magnitude of linkage disequilibrium among sites (Walsh and Lynch 2018, chapters 2–4).

One limitation of single-sample approaches is that most patterns of variation are functions of at least two evolutionary forces. If one desires an estimate of one particular population-genetic parameter, this then requires a preexisting estimate of another key parameter. For example, a common procedure uses silent-site diversity (*π_s_*, obtained as an average from a sample of multiple individuals and nucleotide sites) as an estimator of the equilibrium expectation $4N_{e}u$, which is equivalent to the ratio of the power of mutation to that of drift, where *N_e_* is the effective population size (the inverse of which governs the stochasticity of allele-frequency change) and *u* is the mutation rate per nucleotide site per generation. Extrapolation of an estimate of *N_e_* from *π_s_* requires an estimate of *u*. In the absence of such information, it is often simply assumed that *π_s_* will scale positively with *N_e_*, but this ignores the fact that the mutation rate varies by at least three orders of magnitude across the Tree of Life, scaling nearly inversely with *N_e_* (Lynch et al. 2016; Long et al. 2018). Additional uncertainties associated with single-sample methods include the assumptions of drift-mutation equilibrium and neutrality of the observed polymorphisms.

Similar caveats arise when measures of linkage disequilibrium are used to infer the scaled recombination rate 4*N_e_c*, where *c* is the recombination rate between sites, and when measures of variation at putatively selected sites are used to estimate 4*N_e_s*, where *s* is the strength of selection operating on a nucleotide site. Although some single-sample methods have been derived for reconstructing historical patterns of *N_e_* from the information in site-frequency spectra (e.g., Liu and Fu 2015) or patterns of linkage disequilibrium (Li and Durbin 2011), thereby providing insight into population-size stability, these are unable to estimate *N_e_* within the past several hundred generations, simply because the pool of very young (and rare) alleles is essentially unobservable unless sample sizes are enormous. Moreover, such methods typically retain the assumption of a closed-population structure.

With the field of population genomics now well established, the goal here is to evaluate the limits to the information that can be extracted from temporal sequences of samples of the genomes of multiple individuals, where 10^6^ or so polymorphisms in a sample is in the realm of possibility. An advantage of sequential-sample estimators of *N_e_* and *s* is that they often do not require estimates of mutation or recombination rates (provided these forces are weak relative to those being estimated). In principle, for example, it ought to be possible to estimate the current effective size of a population from observed temporal fluctuations in allele frequencies, provided sample sizes are large enough that the true signal of drift is not overwhelmed by sampling error (Krimbas and Tsakas 1971; Nei and Tajima 1981; Pollak 1983; Waples 1989). However, because such *N_e_* estimators still rely on the assumption of neutrality and a closed-population structure, there is a need for methodology to verify in advance that the molecular markers under consideration are actually consistent with such conditions.

Here, we explore three general issues relevant to the estimation of population-genetic parameters using temporal series of data. First, we develop a simple test for the consistency of the data with neutrality and closed-population structure, as these conditions are ultimately required if temporal fluctuations in allele frequencies are to be reliable indicators of *N_e_*. Second, we explore ways to further evaluate whether polymorphisms at individual nucleotide sites are experiencing significant directional selection, and if not, whether significant fluctuating selection, for example, quasi-neutrality in the sense of Wright (1948) and Kimura (1954) is occurring. Finally, for situations in which the data appear to be consistent with the assumptions of the model, we evaluate the challenges of estimating *N_e_* in large populations by the sequential sampling method.

As the goal is to determine the limits to the ability to estimate current-day population-genetic parameters, it will be assumed throughout that accurate estimates of individual genotypes have been obtained (assured, e.g., with adequate depth of sequencing coverage per site and application of suitable base-call quality screening). All sampling error is therefore associated with the number of surveyed individuals and nucleotide sites. Polymorphic sites will be assumed to be biallelic, as is almost always observed in population samples, although pooling of alleles can be performed with tri- or tetra-allelic sites. As the focus is on populations of relatively large size, it will be assumed that the sampling procedure has minimal effect on the change in population allele frequencies over time. For purposes of presentation, an even temporal distribution of samples will be assumed throughout. The derived expressions for the expected sampling variances of estimates are those for single surveys, but if one had the luxury of performing *η* experimental replications, then the expected sampling variances would be 1/*η* times the reported expressions, and the standard errors should be multiplied by $1/\sqrt{\eta }.$

## Validating the Primacy of Random Genetic Drift

**Fig. 1.**
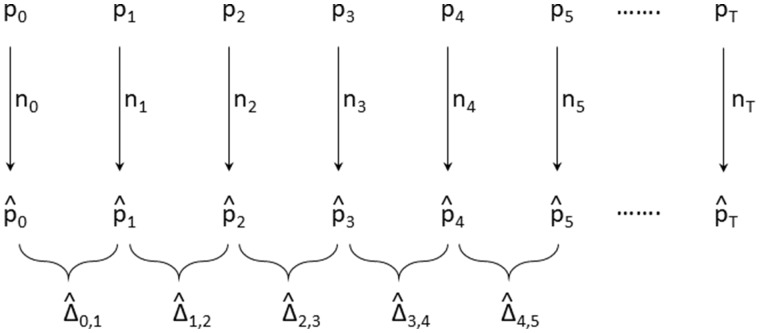
—Sampling scheme for allele frequencies, with *p_i_* denoting the parametric frequency in the population, ${\hat{p}}_{i}$ denoting the estimated frequency after sampling *n_i_* individuals, and ${\hat{\Delta }}_{ij}$ denoting the interval-specific estimated change in frequency.

A critical assumption in the application of temporal methods for estimating *N_e_* is that the series of sequential changes in allele frequencies across sampling points are consistent with a random walk. Consider a temporal series of allele frequencies with population parametric values at nucleotide site *i* equal to $p_{i,0},\;p_{i,1},\ldots ,p_{i,T}$ over *T* equally spaced intervals (e.g., generations) (fig. 1). At each sampling point t=0,…,T, the genotypes are determined for *n_i_*_,__*t*_ individuals (here assumed to be diploid), yielding a series of allele-frequency estimates ${\hat{p}}_{i,0},{\hat{p}}_{i,1},\ldots ,{\hat{p}}_{i,T}$. In the following, we will drop the subscript for nucleotide sites for simplicity unless otherwise noted, and we will assume a constant sample size *n*. From these data, a series of allele-frequency change estimates can be obtained between adjacent samples: ${\hat{\Delta }}_{0,1},{\hat{\Delta }}_{1,2},\ldots ,{\hat{\Delta }}_{T-1,T}$. More generally, allowing for any number of generations separating samples ${\hat{\Delta }}_{j,k}={\hat{p}}_{k}-{\hat{p}}_{j}.$

If random genetic drift is the predominant evolutionary force, because the process has no memory, there should be no correlation between the parametric allele-frequency changes in different intervals, and the interval-specific changes should be random values with expectations equal to zero. The expected variance of change between generation *j* and *j *+* *1 is equal to $\sigma^{2}(\Delta_{j,j+1})=p_{j}(1-p_{j})/(2N_{e}),$ (assuming constant *N_e_* across generations), although as discussed below, additional variance is introduced by sampling of individuals each generation.

There are a number of ways to test for randomness in allele-frequency change. For example, one might evaluate whether the changes in allele frequencies in intervals spaced by a specific number of generations are correlated, which would not be expected if random drift were the predominant evolutionary force (Buffalo and Coop 2019). Even here, however, a number of subtleties can lead to misleading interpretations. First, even under a pure drift model, changes in adjacent intervals will be negatively correlated, owing to the sharing of an intermediate sampling point—if *p_t_* is overestimated by investigator sampling error, then ${\hat{\Delta }}_{t-1,t}$ will be overestimated and ${\hat{\Delta }}_{t,t+1}$ will be underestimated, and vice versa if *p_t_* is underestimated. As this leads to a substantial expected covariance equal to $-p_{t}(1-p_{t})/(2n)$, a comparison of allele-frequency changes in adjacent intervals must be avoided. One might imagine an efficient use involving the pairing of changes separated by single intervals, that is, ${\hat{\Delta }}_{0,1}$ with ${\hat{\Delta }}_{2,3},\;{\hat{\Delta }}_{3,4}$ with ${\hat{\Delta }}_{5,6}$, etc. However, even in this case, owing to the sharing of time points 3, 6, and so on, the expected covariance of pairs of changes has a positive bias ≃p(1−p)/(2nm), where *m *=* T*/3 is the total number of paired intervals. Thus, we now focus on three sampling schemes with minimal to zero bias in the expected values for the covariance in allele-frequency changes under neutrality, the first two focused on the specific behavior of individual sites, and the third involving an aggregate measure over multiple sites.

### Sampling Scheme 1

Given the amount of effort invested in a temporal survey, one will want to use as much of the data as possible, and avoiding the types of adjacent pairings noted above, this might still be accomplished by evaluating the covariance of allele-frequency changes for all pairs of observations separated by single intervals. This can be accomplished by pairing Δ_0,1_ with Δ_2,3_, Δ_1,2_ with Δ_3,4_, Δ_2,3_ with Δ_4,5_, up to $\Delta_{T-3,T-2}$ with $\Delta_{T-1,T}$. One issue with this approach is that some of the Δ values are used twice (e.g., Δ_2,3_), and most still share a sampling point (e.g., Δ_0,1_ and Δ_1,2_), that is, the terms involved in the analysis are still not completely independent. The net result is that the expected covariance is slightly negative, rather than zero.

To see this, note that the unbiased sample covariance estimator for *m* pairs of variables, *x* and *y*, is

(1) $$\text{Cov}(x,y)=\frac{m(\bar{xy}-\bar{x}\cdot \bar{y})}{\left(m-1\right)}.$$

For this particular sampling scheme, m=T−2, so that when *T *=* *5 (six samples in total), *m *=* *3, and the covariance for the site-specific changes is obtained by letting

$$\begin{array}{cc} \bar{xy} & =[(\Delta_{0,1}\cdot \Delta_{2,3})+(\Delta_{1,2}\cdot \Delta_{3,4})+(\Delta_{2,3}\cdot \Delta_{4,5})]/3\\ \bar{x} & =(\Delta_{0,1}+\Delta_{1,2}+\Delta_{2,3})/3\\ \bar{y} & =(\Delta_{2,3}+\Delta_{3,4}+\Delta_{4,5})/3. \end{array}$$

Generalizing to arbitrary *T*, taking expectations of all terms, and noting that all expected cross-products of terms not sharing sampling points are equal to zero (as they share no drift or sampling deviations), the expected covariance for this sampling scheme under neutrality becomes

(2a) $$E[{\text{Cov}}_{1}]=-\frac{E(\delta_{d,2}^{2}+\cdots +\delta_{d,T-3}^{2})}{\left(T-3)(T-2\right)},$$

where $\delta_{d,t}^{2}=p_{t}(1-p_{t})/(2N_{e})$ is the expected squared deviation of allele-frequency change over interval (*t*, *t *+* *1) resulting from genetic drift. Because the expected heterozygosity declines by a factor of 1/(2*N_e_*) per generation, the previous expression reduces to

(2b) $$E[{\text{Cov}}_{1}]=-\frac{p_{0}(1-p_{0})}{2(T-3)(T-2)N_{e}}\cdot \sum_{i=2}^{T-3}\lambda^{i},$$

where $\lambda =1-[1/(2N_{e})].$ However, for populations with $N_{e}\gg T$ (most practical applications), λ≃1.0, and

(2c) $$E[{\text{Cov}}_{1}]≃-\frac{p_{0}(1-p_{0})(T-4)}{2(T-3)(T-2)N_{e}}.$$

This shows that the expected sampling covariance of allele-frequency changes separated by an interval is negative if all intervals are used. Although the absolute bias is expected to be very small in the case of very large populations, the dependency on *N_e_* remains a concern, as this is the parameter that one wishes to eventually estimate.

### Sampling Scheme 2

Now consider the situation in which there is no overlap at all in the use of time points in different pairings, that is, contrasting Δ_0,1_ with Δ_2,3_, Δ_4,5_ with Δ_6,7_, Δ_8,9_ with Δ_10,11_, up to $\Delta_{T-3,T-2}$ with $\Delta_{T-1,T}$. The expected covariance under neutrality is zero with this sampling scheme, although the cost is a substantially longer experiment. For example, with scheme 1, three sets of comparisons can be obtained with *T *=* *5, but this requires *T *=* *11 with scheme 2. A simple expression for the expected sampling variance of the covariance under scheme 2 can be obtained by noting from equation (1),

(3a) $$\sigma^{2}[\text{Cov}(x,y)]={\left(\frac{m}{m-1}\right)}^{2}\{\sigma^{2}(\bar{xy})-2\sigma [\bar{xy},(\bar{x}\cdot \bar{y})]+\sigma^{2}(\bar{x}\cdot \bar{y})\}.$$

Expanding the terms for $\bar{xy},$$\bar{x}$, and $\bar{y},$ noting that $\sigma^{2}(xy)=\sigma_{x}^{2}\cdot \sigma_{y}^{2}$ for two uncorrelated variables each with expectations zero, and that in this case, $\sigma^{2}(\hat{\Delta })=\sigma_{x}^{2}=\sigma_{y}^{2}≃p(1-p)/n$ (because $\hat{\Delta }$, where ^ denotes an estimate, is derived from two samples containing 2*n* genes), leads to the expected sampling standard error for the covariance of changes at a particular site

(3b) $$\sigma [{\text{Cov}}_{2}]=\frac{p_{0}(1-p_{0})}{n\sqrt{m-1}},$$

where *m* is the number of paired Δ comparisons. Averaging over *L* independent sites with different allele frequencies,

(3c) $$\sigma [\bar{{\text{Cov}}_{2}}]=\frac{\bar{p_{0}(1-p_{0})}}{n\sqrt{L(m-1)}}.$$

In contrast, for sampling scheme 1, owing to the nonindependence of data and the associated reduction in degrees of freedom,

(4) $$\sigma [\bar{{\text{Cov}}_{1}}]≃\frac{\bar{p_{0}(1-p_{0})}}{n\sqrt{L(m-1)/2}}.$$

Assuming *T *+* *1 consecutive samples, m=(T+1)/4 for scheme 2, and equation (3c) reduces to

(5) $$\sigma [\bar{{\text{Cov}}_{2}}]=\frac{2\cdot \bar{p_{0}(1-p_{0})}}{n\sqrt{L(T-3)}},$$

and because m−1=T−3 with scheme 1, for equivalent *T*, the sampling error associated with scheme 2 is $2/\sqrt{2}≃1.4\times$ that for scheme 1. Thus, aside from the slight bias, scheme 1 may be viewed as yielding more efficient use of the data. In either case, the preceding expressions indicate that the expected standard error of the test statistic is inversely proportional to the number of individuals sampled per time point (*n*), but only with the approximate square root of the total number of nucleotide sites sampled (≃LT for large *T*).

Note that here and below it is assumed that when estimators are averaged over sites, the vast majority of sites are effectively in linkage equilibrium, as the *L* deployed in the sampling-variance estimators is equivalent to the number of degrees of freedom. This will generally not be a problem if unlinked sites are relied upon. However, studies involving specialized base-population crosses, such as multiparent populations that initiate with high levels of linkage disequilibrium (King and Long 2017), could be especially challenging here. Although the actual degrees of freedom might be approximated through computational comparison of observed sampling variances with those expected with *L* degrees of freedom, this would require prior information on recombination rates. The key point is that, when applied to composite estimators based on multiple loci, all expressions derived herein yield lower-bound estimates of the sampling variance if *L* is used as the number of degrees of freedom.

### Genome-Wide Survey

Whereas the two preceding sampling schemes consider the covariance of change within particular loci over multiple intervals, such approaches are strongly limited by the duration of sampling. An alternative route is to evaluate the average genome-wide covariance of change across *L* sites with a shorter temporal-series duration,

(6) $${\text{Cov}}_{3}=\frac{L\cdot (\bar{\Delta_{ij}\Delta_{ik}}-\bar{\Delta_{ij}}\cdot \bar{\Delta_{ik}})}{L-1},$$

where *j* and *k* denote two time intervals, and the means in the numerator are over all i=1,…,L sites. Under neutrality, assuming the time intervals share no samples, ${\text{Cov}}_{3}$ has expected value zero, and assuming L≫1, sampling standard error

(7) $$\sigma [{\text{Cov}}_{3}]=\frac{\bar{p_{0}(1-p_{0})}}{n\sqrt{L}},$$

identical to equation (3c) with *m *=* *2. Note that this sort of analysis can be performed on any pair of intervals over the sampling period, provided there is no overlap in the sampling points. One might, for example, consider the covariance of change between interval (0,1) and interval (2,3), interval (3,4), etc.

For hypothesis testing, we take advantage of work by Pearson et al. (1929), where the sampling distribution of a covariance for a bivariate normal sampling distribution was derived. Letting ${\hat{\sigma }}_{x,y}$ denote a sample covariance, and *σ_x_*, *σ_y_*, and *ρ* be the parametric standard deviations of *x* and *y*, and their correlation coefficient, and using the standardized variable $v=L{\hat{\sigma }}_{x,y}/[(1-\rho^{2})\sigma_{x}\sigma_{y}]$,

(8a) $$\text{pdf}(v)=\frac{{\left(1-\rho^{2}\right)}^{(L-1)/2}}{\sqrt{\pi }\cdot 2^{(L/2)-1}\cdot \Gamma [(L-1)/2]}\cdot \;\text{exp}(\rho v)\cdot v^{(L/2)-1}\cdot K_{(L/2)-1}(v),$$

where Γ(x) is the gamma function, and $K_{(L/2)-1}(v)$ denotes a modified Bessel function of the second kind. For the special case in which *ρ *= 0 (our null hypothesis), Pearson et al. (1929) found that the preceding expression can be closely approximated (perfectly to the first four moments) with

(8b) $$\text{pdf}(v)=\frac{\Gamma [(L+4)/2]}{\Gamma [(L+3)/2]\sqrt{\pi (L^{2}-1)}}{\left(1+\frac{v^{2}}{L^{2}-1}\right)}^{-(L+4)/2}.$$

In our case, $v=Ln{\hat{\sigma }}_{x,y}/[p_{0}(1-p_{0})],$ and with L≫1, equation (8b) simplifies further to

(8c) $$\text{pdf}(v)≃\frac{1}{\sqrt{2\pi L}}\cdot \;\text{exp}\;\left(-\frac{v^{2}}{2L}\right),$$

showing that the standardized covariance measure *v* is normally distributed with variance *L*.

This suggests a relatively simple test for random allele-frequency changes across different intervals. Let $\hat{\sigma }(\Delta \prime )$ be the covariance of standardized allele-frequency changes for pairs of intervals, with the change at each site being normalized by the expected standard deviation of changes, that is, using ${\hat{\Delta }}_{j_{0},j_{1}}^{\prime }=({\hat{p}}_{j_{1}}-{\hat{p}}_{j_{0}})/\sqrt{\hat{p}(1-\hat{p})/n}$ as the standardized change between the time points *j*_0_ and *j*_1_ for interval *j*, with $\hat{p}$ being the average allele frequency over the two time samples at the site, with a similar definition for interval *k*. Because $\hat{\sigma }(\Delta \prime )=v/L$, it follows that

(8d) $$\text{pdf}[\hat{\sigma }(\Delta \prime )]≃\frac{1}{\sqrt{2\pi /L}}\cdot \;\text{exp}\;\left\{-\frac{{\left[\hat{\sigma }(\Delta^{\prime })\right]}^{2}}{2/L}\right\},$$

that is, under the null hypothesis, the sample covariance of standardized allele-frequency changes has expectation zero and variance 1/L. (Again, as noted above, if the assayed loci are linked, *L* needs to be replaced with the appropriate degrees of freedom.) A test for random change is then achieved by comparing the absolute value of $\hat{\sigma }(\Delta \prime )$ with the critical two-tail cutoff points of the normal distribution. If the data are consistent with a model of pure drift, for large *L*, there is a 5% probability that the absolute value of $\hat{\sigma }(\Delta \prime )$ will exceed $1.96/\sqrt{L}$ by chance, and a 1% probability that it will exceed $2.58/\sqrt{L}$ by chance. Observed values of $\left|\hat{\sigma }(\Delta \prime )\right|$ beyond these critical points imply that sampling heterogeneity problems, migration, and/or selection have contributed to the average observed pattern of change.

## Estimation of the Mean and Variance of Selection Intensity

Results from hundreds of single-sample studies in molecular population genetics suggest that the intensity of directional selection operating at the single-nucleotide level is often on the order of the reciprocal of *N_e_* or a factor several-fold larger. Selection coefficients at the nucleotide level >0.01 are exceedingly rare in studies of natural populations, and as these only induce an ∼1% change in allele frequency per generation, the challenges in estimating selection at the DNA level with temporal data are clear. An additional issue (aside from possible contributions from nonselective forces) is that temporal changes in allele frequencies may result from direct selection on the nucleotide site of interest or indirectly from selection operating on adjacent sites in linkage disequilibrium. Thus, the best that we can hope to achieve with a temporal survey is a measure of the net strength of selection operating on a site.

One way forward is to note that in the absence of frequency-dependent selection, and assuming additive fitness effects, the expected change in allele frequency over an interval of *t* time units is

(9a) $$\zeta_{t}=\zeta_{0}-\mu_{s}t,$$

where

(9b) $$\zeta_{t}=\ln\left(\frac{1-p_{t}}{p_{t}}\right),$$

and *μ_s_* is the average interval-specific selection coefficient over the entire sampling period (Crow and Kimura 1970). It follows that the slope of a regression of *ζ_t_* on time provides an estimate of *μ_s_* for the allele designated by frequency *p*. This estimator is only defined when the allele-frequency estimate is $0<{\hat{p}}_{t}<1$ at all sample points. In principle, the selection coefficient may also fluctuate in time, one case of special interest being quasi-neutrality, wherein the long-term average *s* is equal to zero with the interval-specific magnitudes of selection wandering randomly around this mean with temporal variance $\sigma_{s}^{2}$ (Wright 1948; Kimura 1954).

### Estimation of Mean Selection Coefficients

**Fig. 2.**
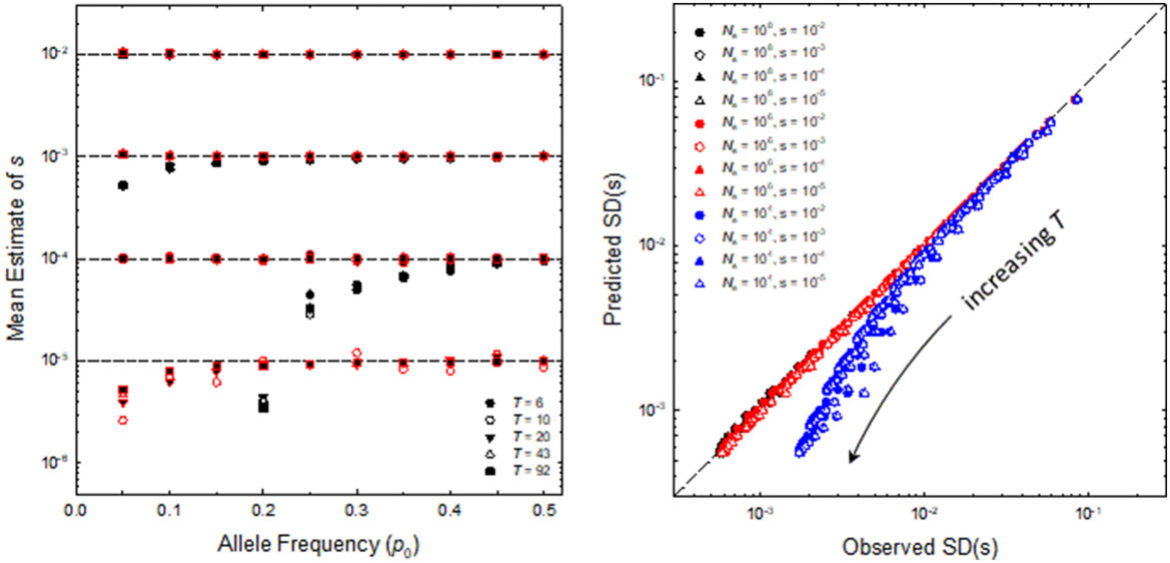
—(Left) Mean estimates of the selection coefficient *s* obtained from the least-squares regression approach. Each point is the average of the results from 10^7^ simulations based on Wright–Fisher allele-frequency dynamics incorporating selection and drift, followed by random sampling of *n *=* *100 diploid individuals at each sampling point. Black symbols are for effective population size $N_{e}=10^{4}$, and red for $N_{e}=10^{6}$, and results are reported for a range of starting allele frequencies, *p*_0_. The horizontal dashed lines denote the expectations for four evaluated selection coefficients (with temporal variance, $\sigma_{s}^{2}$, equal to zero), and the different symbols denote experiments of different durations (*T*). (Right) Sampling standard deviations for estimates of *s* for the case of $\sigma_{s}^{2}=0$, from simulations as noted above for three values of *N_e_*, four of *s*, and a sample size of 100, compared with the theoretical expectation, equation (10). The diagonal dashed line denotes points of perfect agreement, and many symbols cannot be seen as they overlie each other on this line.

An efficient means of estimating *μ_s_* for a nucleotide site is to perform a least-squares regression of *ζ_t_* on time. Allowing for both selection and drift in a Wright–Fisher framework, followed by random sampling, computer simulations indicate that the regression coefficients provide unbiased estimates of *μ_s_* over reasonable sample sizes and allele frequencies, so long as selection is strong enough to dominate random genetic drift (fig. 2, left). Negative bias occurs, independent of the experimental duration and sample size, when $N_{e}sp_{0}<1,$ consistent with the view that selection operates in nearly deterministic fashion only after an allele frequency exceeds $1/(N_{e}s)$ (Walsh and Lynch 2018, chapter 7), as assumed in equation (9b). In principle, a more elaborate expression for allele-frequency change that allows for the influence of drift might be developed, but this would require an estimate of $N_{e}.$

An expression for the expected sampling variance of the regression coefficient (the sample estimate $\hat{s}$ of the population parameter *μ_s_*), the steps of which can be found in the Appendix, is

(10) $$\sigma^{2}(\hat{s})≃\left[\frac{6}{\left(T-1)(T+1\right)}\right]\left[\frac{1}{Tnp_{0}(1-p_{0})}+\sigma_{s}^{2}\right],$$

where $\sigma_{s}^{2}$ is the among-generation variance in *s*. This expression provides an essentially unbiased estimate of the sampling variance obtained in computer simulations, for a wide range of experimental durations (*T *=* *5–90), sample sizes (*n *=* *100–1,000), full range of allele frequencies ($p_{0}=0.05$–0.50), and a range of selection coefficients ($s=10^{-5}$ to $10^{-2}$), provided $N_{e}>10^{5}$ (fig. 2, right). For weak selection and long experimental durations, equation (10) somewhat underestimates the sampling variance because it does not account for the cumulative effects of random genetic drift.

In practical applications, one would ordinarily accept the estimate of the sampling variance of the regression coefficient from direct statistical analysis, but the expectation given by equation (10) provides insight into the optimal design of sampling schemes for estimating $\mu_{s}.$ Regardless of the average strength of selection, provided $T\sigma_{s}^{2}$ is small relative to the sampling variance of *ζ*, for *T *>* *10 or so, the sampling variance of $\hat{s}$ is inversely related to the product of the sample size and the cube of the number of temporal samples. Thus, for a fixed investment in the total amount of genotyping that can be done, which is proportional to *Tn*, there is a very strong premium on extending the experiment in time, as the expected standard error of $\hat{s}$ will be inversely proportional to 1/T.

One can go further and consider the overall design necessary to detect a nucleotide with mean selection coefficient $\mu_{s}.$ Assuming $\sigma_{s}^{2}$ is small relative to the sampling-error term in equation (10), which seems likely for most reasonable scenarios, the minimum sampling variance reduces to $≃6/[T^{3}np_{0}(1-p_{0})].$ To detect a selection coefficient at the 5% significance level, one then requires $24/[T^{3}np_{0}(1-p_{0})]<\mu_{s}^{2}.$ The greatest power is achieved with high allele frequencies, so letting $p_{0}=0.5,$ the critical value for detection in this case is $T^{3}n=96/\mu_{s}^{2}$, which implies $T^{3}n>10^{6}$ for $\mu_{s}=0.01,$ and $>10^{8}$ for $\mu_{s}=0.001.$ Assuming a moderate sample size of *n *=* *100, the critical experimental durations in these two cases become 21 and 100 consecutive generations of allele-frequency estimation. For a rarer allele with frequency $p_{0}=0.1,$ these critical values become 2.8× larger.

The key point here is that when selection is weak, as is generally the case at the nucleotide level, its detection using temporal series of data demands very long surveys. Increasing the sample size helps, but in expanding *n* to 1,000, the above critical *T* values decline by only ∼50%, and temporal variance in the selection coefficient will make such an enterprise more demanding. If one simply desires an estimate of the average absolute value of *μ_s_* over a large sample of sites (e.g., particular sites within codons at particular frequencies), the sampling variance of the mean estimate is given by equation (10) divided by the number of sites jointly evaluated.

How much accuracy in estimation would be lost if one simply relied upon a two-point estimate of $\hat{s},$

(11) $${\hat{s}}_{2}=\frac{\zeta_{0}-\zeta_{T}}{T},$$

rather than using allele-frequency estimates at each time point? From Lynch (1987), the expected sampling variance of the two-point estimate is

(12) $$\sigma^{2}({\hat{s}}_{2})=\frac{1}{T^{2}n}\left[\frac{1}{p_{0}(1-p_{0})}+\frac{1}{p_{T}(1-p_{T})}\right].$$

Assuming $\sigma_{s}^{2}≃0$ and T≫1, the ratio of the sampling variance in this case relative to that with a full survey is

(13) $$\frac{\sigma^{2}({\hat{s}}_{2})}{\sigma^{2}({\hat{s}}_{T})}≃\frac{T}{6}\left[1+\frac{p_{0}(1-p_{0})}{p_{T}(1-p_{T})}\right].$$

The minimum improvement gained by the full survey is therefore a reduction in the standard error of the estimate $\hat{s}$ by a factor of ${\left(T/6\right)}^{1/2}$, that is, 2× with *T *=* *24, and 4× with *T *=* *96. In the limit of weak selection and/or short survey duration, such that $p_{0}(1-p_{0})≃p_{T}(1-p_{T}),$ the inflation in sampling variance with the simpler method is a factor of ≃T/3, whereas as the allele frequency approaches loss or fixation, that is, $p_{T}(1-p_{T})\to 0,$ the inflation factor can exceed *T*.

Equation (10) can also be used to evaluate the consequences of more intermediate sampling schemes. Rather than sampling each of (T+1) consecutive generations, one could skip various generations, so that the duration of each sampling interval is *D* (rather than 1 or *T*) generations. The expected sampling variance of $\hat{s}$ is then obtained by dividing equation (10) by *D* and substituting the number of multigenerational time intervals, T′, for *T*. For *T* divisible by *D*, the inflation in the sampling standard error is $≃\sqrt{D}.$ As an example, for a full survey with *T *=* *49 and *D *=* *1, from equation (10), the expected sampling variance is $≃0.000050/[np_{0}(1-p_{0})].$ Keeping $np_{0}(1-p_{0})$ constant, and reducing the overall effort by half by skipping single generations, T′=24 and *D *=* *2, and the expected inflation of the standard error of $\hat{s}$ is 1.5×. With T′=12 and *D *=* *4 (skipping periods of three generations), the expected inflation is 2.1×, and with T′=6 and *D *=* *8, the expected inflation is 2.6×. From equation (13), the expected inflation in the extreme case of sampling at just the starting and ending points (equivalent to a 25-fold reduction in effort) is ∼$\sqrt{T/3}=2.9.$ The key point here is that, for a given total survey duration, the improvement in the accuracy of estimation of *μ_s_* with increased frequency of sampling is relatively small compared with the increase in effort.

### Estimator for the Variance of Selection Coefficients

To estimate the variance in true selection coefficients among generations, $\sigma_{s}^{2}$, we note that the estimated selection coefficient in interval *i* can be partitioned as

(14) $${\hat{s}}_{i}=s_{i}+e_{i},$$

where *s_i_* is the true selection coefficient operating on the site in generation *i*, and *e_i_* is the estimation error resulting from finite sample size. From this, it follows that an estimator of the variance in *s* among intervals is

(15) $${\hat{\sigma }}_{s}^{2}=\text{Var}(\hat{s})-\text{Var}(e),$$

where Var(e) is the average sampling variance of the *s_i_*. Here, we focus on the most fine-grained sampling scheme of *T* intervals of single-generation duration, as this will yield estimates of $\sigma_{s}^{2}$ with the highest degree of accuracy. To obtain an estimate of $\text{Var}(\hat{s})$ unfettered by nonindependence problems, we focus on the variance of *s_i_* estimates obtained for nonoverlapping time intervals, for example, (0,1),(2,3),…,(T−2,T−1). For even *T*, this yields τ=T/2 estimates, with

(16) $$\text{Var}(\hat{s})=\frac{1}{\tau -1}\sum_{i=1}^{\tau }{\left({\hat{s}}_{i}-\bar{s}\right)}^{2}$$

being the estimated variance among interval-specific sample estimates of *s_i_*. From equation (11) with *T *=* *1, estimates of *s_i_* based on adjacent generations are simply equal to the difference in estimates of *ζ* across the interval.

To estimate the average sampling variance, we utilize all of the data but allow for nonindependence of adjacent *s_i_* estimates (owing to allele frequencies at shared time points),

(17) $$\text{Var}(e)=\frac{1}{T}\sum_{i=1}^{T}\sigma^{2}({\hat{s}}_{i})-\frac{2}{T(T-1)}\sum_{i<j}^{T}\sigma ({\hat{s}}_{i},{\hat{s}}_{j})$$

(from Lynch and Walsh 1998, p. 845). Note that the second term, which accounts for nonindependent estimates of the selection coefficient, has nonzero entries only for pairs of estimates in adjacent intervals. From Lynch (1987, eq. 12),

(18) $$\begin{array}{c} \sigma^{2}({\hat{s}}_{i})=\phi_{i-1}+\phi_{i}\\ \sigma ({\hat{s}}_{i},{\hat{s}}_{i+1})=-\phi_{i} \end{array}$$

with $\sigma ({\hat{s}}_{i},{\hat{s}}_{j})=0$ for j≠i+1, and $\phi_{i}=1/[2n_{i}{\hat{p}}_{i}(1-{\hat{p}}_{i})],$ where *n_i_* is the number of diploid individuals in the *i*th sample (the two being removed in the case of haploidy). Substituting into equation (17), for the situation in which a string of *T* consecutive estimates of *s_i_* is available,

(19) $$\text{Var}(e)=\frac{1}{T}\left(\phi_{0}+\phi_{T}+\frac{2T}{T-1}\sum_{i=2}^{T}\phi_{i-1}\right).$$

Solving equations (16 and 19), and applying to equation (15) then provides an estimate of the variance in the selection coefficient for a nucleotide site.

**Fig. 3.**
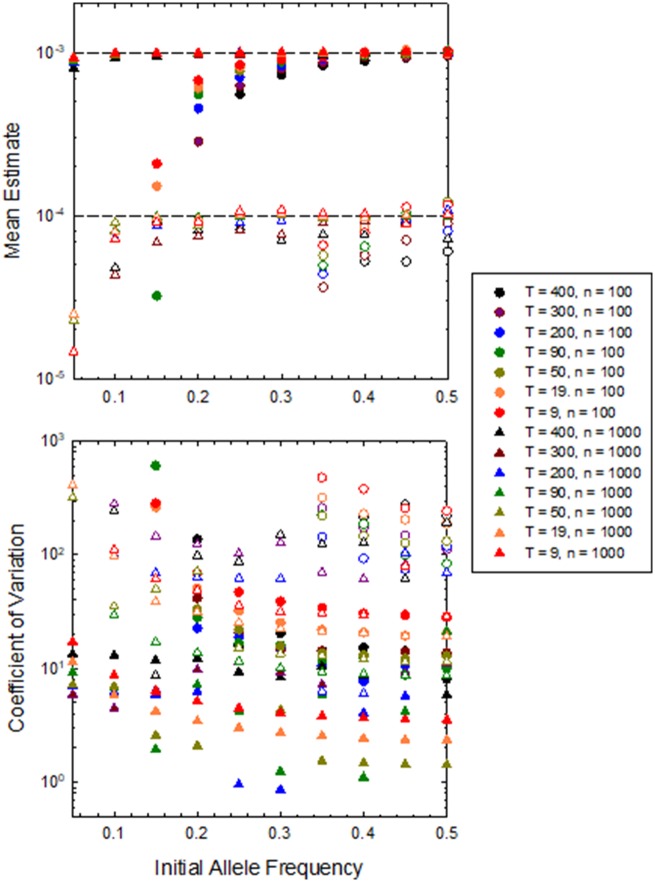
—Mean and CV of estimates of $\sigma_{s}^{2}$ for series of samples taken at *T *+* *1 consecutive time points, each involving sample sizes of *n *=* *100 or 1,000 diploid genomes. Results are given for a range of initial allele frequencies, each based on 10^6^ simulations with an effective population size of 10^8^ individuals, ensuring essentially no genetic drift on the time scale of the analyses, and mean selection coefficient $\mu_{s}=0.0.$ Closed points refer to situations in which $\sigma_{s}^{2}=10^{-3}$, whereas open points are for $\sigma_{s}^{2}=10^{-4}.$ Data points are excluded for some cases at low allele frequencies where the mean estimates of $\sigma_{s}^{2}$ were negative.

Computer simulations incorporating generational episodes of selection and random genetic drift, with $\mu_{s}=0,$ were used to determine the bias and sampling error associated with this estimator of $\sigma_{s}^{2}$ (fig. 3). Two points are immediately apparent. First, the estimates for $\sigma_{s}^{2}$ tend to be downwardly biased, particularly when initial allele frequencies are low and sample sizes are on the order of 100 or smaller. This bias becomes negligible when sample sizes are as large as 1,000. However, even in the latter case, and even for the long experimental durations illustrated, an unbiased estimate of $\sigma_{s}^{2}$ cannot be achieved if $\sigma_{s}^{2}<10^{-4}.$ Given that the latter implies a standard deviation of *s* of 0.01, which may be beyond what operates at most nucleotide sites, the implication is that achieving accurate estimates of $\sigma_{s}^{2}$ at single-nucleotide sites is nearly unattainable without enormous sample sizes and survey durations.

Second, of even greater concern is the coefficient of variation (CV) of estimates of $\sigma_{s}^{2}$, which is virtually always >1.0 and often as high as 500. With a sampling CV of 1.0, if one wanted an average estimate of $\sigma_{s}^{2}$ pooled over sites to have a standard error <0.1 of the mean, 100 sites would need to be pooled, and with a per-site sampling CV of 500, this same level of accuracy would require the pooling of 25,000,000 sites.

## Estimation of Short-Term *N_e_*

The effective size of a population (*N_e_*) governs a wide range of evolutionary features, including the probability of fixation, levels of standing molecular variation, patterns of linkage disequilibrium, and the range of selection coefficients of mutant alleles that are effectively neutral (Charlesworth 2009; Walsh and Lynch 2018). Unfortunately, *N_e_* is also an exceptionally difficult population-genetic parameter to estimate directly. Almost all direct approaches rely on estimates of allele-frequency change, which invariably have very high sampling variances. Nonetheless, as studies of temporal changes in allele frequencies have become increasingly common, there has been a resurgence of interest in using such data to estimate *N_e_*.

The motivation for the general approach is that random genetic drift generates stochastic changes in neutral allele frequencies to a degree determined by $1/(2N_{e})$ in diploids (and $1/N_{e}$ in haploids). With suitable correction for the additional variation in allele-frequency estimates induced by sampling, observed divergence can then be used to infer *N_e_* provided the variation evaluated is neutral (Krimbas and Tsakas 1971). Often, a method of moments is used to infer *N_e_* from the observed allele-frequency changes and the sample sizes (Nei and Tajima 1981; Pollak 1983; Waples 1989), although likelihood-based procedures have also been suggested (Wang 2001; Bollback et al. 2008; Malaspinas et al. 2012; Hui and Burt 2015; Ferrer-Admetlla et al. 2016). Almost all applications have been confined to small populations, with *N_e_* typically well below 5,000. In this case, with suitable numbers of markers and time spans between samples, reasonable *N_e_* estimates (often within 10% of the expected values) are obtainable.

Because most natural populations are thought to have *N_e_* in the range of 10^4^ to 10^9^ (Lynch 2007), it is desirable to know in advance the joint influence of the numbers of sampled nucleotide sites, individuals, and generations separating samples on the ability to achieve a perceptible signal from drift. Is there an important tradeoff between the size of samples and the duration of sampling? Are there significant advantages of incremental sampling, as opposed to simply sampling at the starting and ending points of a survey? Do the frequencies of alleles at the sampled nucleotide sites have a substantial degree of influence?

To answer these questions, we proceed under the assumption that the investigator has settled on a set of molecular markers that suitably fulfill the expectations under neutrality based, for example, on the types of tests suggested above. The key indicator variable for estimating *N_e_* is then

(20) $$\phi_{\mathrm{ijk}}={\hat{F}}_{\mathrm{ijk}}-\frac{1}{2n_{ij}}-\frac{1}{2n_{ik}},$$

where *i* denotes the nucleotide site, *j* and *k* denote two sampling times, and *n_ij_* and *n_ik_* are the associated numbers of sampled individuals (here assumed to be diploid). ${\hat{F}}_{\mathrm{ijk}}$ is a normalized measure of the observed allele-frequency change between the two time points,

(21) $${\hat{F}}_{\mathrm{ijk}}=\frac{{\left({\hat{p}}_{ij}-{\hat{p}}_{ik}\right)}^{2}}{{\bar{p}}_{i}(1-{\bar{p}}_{i})},$$

where ${\hat{p}}_{it}$ is the estimated allele frequency at time *t*, and ${\bar{p}}_{i}=({\hat{p}}_{ij}+{\hat{p}}_{ik})/2.$ In essentially all of the following analyses, simulations show that the ratio of the variance of individual ${\hat{F}}_{\mathrm{ijk}}$ estimates to the squared average value is in the range of 1.90–2.00, as expected for a $\chi^{2}$-distributed variable (Nei and Tajima 1981). In the following, it will be assumed that the sample sizes are constant across time periods, so that $n=n_{ij}=n_{ik}.$

The logic underlying the use of ${\hat{F}}_{\mathrm{ijk}}$ is that the expected value of *ϕ_ijk_* is very close to *t*/(2*N_e_*) provided the number of generations separating the two samples (*t*) is much smaller than $2N_{e}$ (Nei and Tajima 1981; Pollak 1983; Waples 1989), which will generally be the case for a study of natural populations. The resultant method-of-moments estimator of *N_e_* from two temporal samples is then

(22) $${\hat{N}}_{e}=\frac{t}{2{\bar{\phi }}_{i}},$$

where ${\bar{\phi }}_{i}$ is the average value of *ϕ_i_* over all sites. (This averaging of the denominator term to obtain a single estimate of *N_e_* rather than obtaining an estimate for each site and then averaging minimizes the spurious sampling variation that can occur with individual ratios.)

Pollak (1983; see also Waples 1989) has usefully found that the expected sampling variance of ${\hat{N}}_{e}$ is

(23a) $$\sigma^{2}({\hat{N}}_{e})≃\left(\frac{8N_{e}^{4}}{L}\right)\left[\frac{1}{{\left(2N_{e}\right)}^{2}}+\frac{1}{tN\tilde{n}}+\frac{1}{{\left(t\tilde{n}\right)}^{2}}\right],$$

where *L* is the number of sampled polymorphic sites (assumed to be independent), $\tilde{n}$ is the harmonic mean of the sample sizes (equal to *n* in all that follows), and *N* is the actual number of breeding adults (typically much larger than *N_e_*; Walsh and Lynch 2018). Although an approximation, this formula provides immediate insight into the determinants of the precision of *N_e_* estimates by the temporal method. Because we expect $t\tilde{n}\ll 2N_{e}<N$, equation (23a) reduces to

(23b) $$\sigma^{2}({\hat{N}}_{e})≃\left(\frac{2}{L}\right){\left(\frac{2N_{e}^{2}}{t\tilde{n}}\right)}^{2}.$$

The coefficient of sampling variation of ${\hat{N}}_{e}$ (the ratio of the standard deviation of estimates to the expected value) is then

(24) $$\text{CV}({\hat{N}}_{e})≃\frac{2N_{e}\sqrt{2/L}}{t\tilde{n}}.$$

The remaining issue is whether the preceding formulae, all of which involve approximations, do indeed yield reasonable estimates of *N_e_* and its sampling variance. This was evaluated by simulating three sources of binomial sampling of allele frequencies: random genetic drift between points *j* and *k* and individual sampling at the two time points. For any particular set of conditions, starting with a known allele frequency *p_j_*, drift involved the random sampling of *N_e_* = *N* individuals to generate *p_k_*, and then from these two true population values, the estimated frequencies ${\hat{p}}_{j}$ and ${\hat{p}}_{k}$ were obtained. For any particular set of $N_{e},$*L*, *t*, and *n*, the mean and variance of ${\hat{N}}_{e}$ (estimated by eq. 22) was obtained over a full range of allele frequencies. Over a range of $N_{e}=10^{3}$ to 10^6^, the preceding theory is highly consistent with the results from simulations—equations (22 and 23b) give essentially unbiased estimates of the mean and variance of *N_e_* estimates, provided $tn\sqrt{L}\gg 5N_{e}$.

Equation (24) indicates that changing the survey duration *t* or the sample size *n* by the same factor has an equivalent effect on the accuracy of *N_e_* estimates by the temporal method. Such a scaling provides a logical basis for the optimal sampling design constrained by economic considerations and practicalities with respect to long-term sampling. The CV scales with $N_{e}/t$ because the variance of allele-frequency change resulting from drift (the signal that we wish to detect) depends on the reciprocal of this quantity. On the other hand, the CV scales only with the inverse of the square root of the number of loci sampled, which assuming complete genome sequencing is an absolute limit that cannot be modified by sampling.

This sampling theory highlights the difficulties in estimating *N_e_* in large populations by direct observations of allele-frequency changes. Nonsensical estimates of *N_e_* can be expected if $tn\sqrt{L}<5N_{e}$, as then $\text{CV}({\hat{N}}_{e})$ exceeds 0.5, and even negative estimates will be likely. Suppose one desires to reduce the CV of an *N_e_* estimate to 0.1. According to equation (24), this requires $0.035tn\sqrt{L}>N_{e}.$ Further supposing the luxury of $L=10^{6}$ sites, the critical point reduces to $35tn>N_{e},$ which means that with $N_{e}=10^{6}$, *tn* must exceed 28,600, a steep task—sample sizes of nearly 3,000 if one were to rely on a temporal duration of 10 generations. On the other hand, with $N_{e}=10^{4},$*tn* need only exceed 290, an achievable situation with today’s sequencing costs (e.g., two samples of size 60, five generations apart).

The preceding estimator is based on a simple two-point comparison, and one might imagine that more resolution would be achievable by sampling at multiple intervals during time span *t*. There is, however, reason to expect a minimum payoff of such additional monitoring, the primary being that there is such a strong covariance in allele frequencies across generations that most of the information is contained within the contrast between starting and ending points (Hill 1972; Lynch 1987). Indeed, an analysis of a multigenerational maximum-likelihood (ML) procedure for estimating *N_e_* from temporal data (Hui and Burt 2015, table 1) shows that for the two-sample situation, the method-of-moments and ML procedures have almost identical sampling variance, and whereas the method of moments yields higher sampling variance when extended to three samples (over an equal total time span), the sampling error with the ML method is essentially identical whether two or three samples are applied. Such behavior can be understood from the structure of equation (23b), which shows that the sampling variance of *N_e_* scales inversely with *t*^2^. Subdivision of *t* into *x* smaller episodes of equal length *τ* (t=xτ) yields more independent estimates of *N_e_*, which reduces the sampling variance linearly in *x*, but the sampling variance for each episode increases quadratically, the end result being that the sampling variance scales as $∼1/(x\tau^{2})=x/t^{2}.$

To evaluate this matter more formally, allowing for larger *N_e_*, the above kinds of analyses were performed three ways with six samples (providing five intervals of data): 1) using the simple starting and ending points, 2) performing two two-generation analyses (intervals 0–2, and 3–5), and 3) performing three single-generation analyses (intervals 0–1, 2–3, and 4–5). In the limit of $N_{e}\gg tn,$ the preceding argument suggests that the sampling standard deviation of *N_e_* for these three schemes will scale approximately as 1.0:1.4:1.7. The simulation results indicate that the increase in the sampling standard deviation is reasonably consistent with the scaling suggested above, but that the use of multiple estimates also leads to downward bias in the mean estimate. Both features may be a consequence of the positive covariance between seemingly independent samples. An allele-frequency change resulting from drift causes a correlated change in the denominator of consecutive applications of equation (21) via the effects on heterozygosity at the site. In principle, computationally intensive ML techniques might be constructed to alleviate this problem, but taken together, these and prior analyses suggest that there are minimal advantages to doing so.

## Discussion

To ease the presentation, the formulations developed above assumed a setting in which populations are sampled on a timescale equal to generation length but are readily extended to longer intervals. For example, if the sampling interval is equivalent to *D* generations, to account for the temporal accrual of selection effects, the estimated $\bar{s}$ should be divided by *D*, and the estimated variance of *s* should be divided by *D*^2^. Likewise, the estimated *N_e_* would need to be multiplied by *D*, as the effects of drift accumulate linearly with the number of generations. The expressions for the standard errors of these estimates would all have to be scaled in the same way, leaving the coefficients of sampling variance unchanged. There are, of course, alternative sampling schemes such as the fortuitous acquisition of DNA samples from single individuals at uneven intervals in the past. However, these do not easily lend themselves to the sampling theory outlined above, as there is additional uncertainty on the number of generations between intervals and even on lines of descent.

Our results demonstrate that although population genomics offers opportunities for obtaining relatively accurate estimates of previously elusive but key population-genetic parameters, the sampling requirements for achieving such ends remain substantial. As one example, Karasov et al. (2010) have suggested that the current effective population size of *Drosophila melanogaster* exceeds $10^{8},$ more than an order of magnitude larger than prior estimates. If one wished to show by direct estimation that *N_e_* is significantly >10^7^ by an order of magnitude, a standard error of the estimate of *N_e_* of about 45,000,000 would be required. From equation (20b), generously assuming 10^6^ informative neutral sites, this requires that the product of the duration of the survey (*t*, in generations) and the sample size at each point (*n*) exceed $6\times 10^{5}$, which is equivalent to fully genotyping 60,000 individuals at two time points separated by 10 generations. Although such an extreme might be possible in the not too distant future by pooled population sequencing to ∼100,000× depth of coverage, pooled sequencing is compromised by sequencing errors that obscure small frequency changes, as well as by likely variation in the contributions of individuals of different sizes to the pooled DNA. Thus, estimation of large *N_e_* almost certainly requires information on individual genotypes, necessary for factoring out contributions from sequencing errors (Lynch et al. 2014; Maruki and Lynch 2015).

Although the methods outlined above involving temporal covariance of allele-frequency changes provide a formal basis for testing whether temporal series of data are consistent with a random-walk model, it should be noted that violations of the null model need not imply the action of selection. For example, in nonequilibrium situations, persistent migration into a sampling site can lead to consistent directional changes in allele frequencies across intervals. One of the most likely problems, perhaps nearly unavoidable in natural settings, is the presence of spatial heterogeneity within populations. Owing to physical limitations on all organisms, nonrandom dispersal can be expected to be the rule even in populations uninfluenced by migration. Even if neutral, microlocale (spatial) heterogeneity in allele frequencies will manifest as temporal heterogeneity in a series of samples that do not fully account for such structure. In a test tube of microbes, such microheterogeneity might be eliminated by thoroughly mixing the sample before sampling, but this is essentially impossible for natural populations. Given that the shifts in allele frequency generated by random genetic drift are very small when *N_e_* is large, even a small amount of any of these kinds of biases can be problematical.

Assuming an absence of these kinds of problems, Buffalo and Coop (2019) suggested that genome-wide patterns of selection can be inferred indirectly by estimating the covariance in temporal allele-frequency change of neutral markers. Their proposed measure is closely related to the genome-wide covariance method outlined above, except that they normalize the measures of frequency change by dividing by p(1−p). The motivation of this method is the idea that the expected covariance of allele-frequency change is zero in the absence of linked selection, and that when the latter prevails, alleles with larger frequency changes consistently exhibit such behavior across intervals because they happen to be stochastically associated with linked variants with greater fitness effects. The magnitude of such associations depends on the recombination rate among sites and on the time interval between surveys.

It is difficult to project the likely values of the scaled covariance of frequency change with linked selection in natural populations, but preliminary calculations by Buffalo and Coop (2019) suggest that values well below $10^{-3}$ may be common. This could present substantial challenges. Rearranging equation (7) indicates that the expected standard error of the temporal covariance of change in the null situation of no linked selection is $≃1/(n\sqrt{L})$, where *L* is the number of polymorphic sites in the analysis. Assuming a sample size of n=100, then if an observed measure of standardized covariance is $10^{-3}$, rejection of the null hypothesis at the 5% level would require *L *>* *400, and for covariances of $10^{-4}$ and $10^{-5}$, the minimum number of sites grows to 40,000 and 4,000,000, respectively. These constraints are generous in that they assume unlinked loci. If analyses were restricted to bins of strongly linked region of markers, as might be the case in searches for chromosomal regions under various levels of selection, to a first-order approximation, L≃1, and the expected standard error is ≃1/n. The minimum sample size for rejecting the null hypothesis is then on the order of twice the reciprocal of the observed measure of covariance of change. Thus, aside from the physical limitations of obtaining completely random samples, the method of Buffalo and Coop (2019) appears to be extremely demanding with respect to required sampling effort.

There is also an issue with the view that linked selection will always generate positive covariance of allele-frequency change across generations. Even assuming that the selection coefficients of all polymorphisms remain constant in time, it can be seen from equations (9a and 9b) that for any strength of selection, there is an inflection point in the rate of allele-frequency change at *p *=* *0.5 such that on opposite sides of this point the expected rate of change (although always of the same sign) will go from an acceleration to a deceleration phase. This behavior, which is a simple consequence of the frequency dynamics of selected alleles being proportional to p(1−p), means that even with constant selection, marker alleles in different frequency classes are expected to yield different covariances of frequency change ranging from positive to near-zero to negative. The extent to which such complications will obscure the expected signature of linked selection will depend on the site-frequency spectrum of selected alleles and the relative incidence and magnitude of positive and purifying selection. This raises questions about the interpretation of the covariance of allele-frequency change, including whether the neutral expectation of zero overall covariance of change is also likely to arise emerge under some conditions of selection.

Computationally intensive methods involving likelihood and Bayesian frameworks are becoming increasingly popular routes to estimating population-genetic parameters such as *N_e_* and *s*, separately or as the product $N_{e}s$ (Bollback et al. 2008; Malaspinas et al. 2012; Foll et al. 2015; Ferrer-Admetlla et al. 2016). So far, the utility of these methods has been primarily confined to situations involving very small *N_e_* ($<10^{4}$) and large *s* (>0.05), conditions that do not commonly occur in natural populations of cellular organisms. A potential advantage of likelihood methods that arises with large $N_{e}s$ or $t/N_{e}$ is that these conditions raise the possibility of allele-frequency estimates of 0.0 or 1.0, especially if sample sizes are small. Such extremes lead to undefined method-of-moment estimators but can in principle be factored into likelihood functions that attempt to account for the full sampling distribution of alleles. Nevertheless, the latter types of methods do not supersede those outlined above or the statistical limitations that we have highlighted. Indeed, even when *s* is estimated in a Bayesian framework, the background *N_e_* is still frequently estimated with a genome-wide application of the method of moments, equations (20–22), often without prior testing for the neutral behavior of the underlying polymorphisms.

## Appendix

### Sampling Variance of the Mean Selection Coefficient

As noted in the text, letting

(A1a) $$\zeta_{t}=\ln\left(\frac{1-p_{t}}{p_{t}}\right),$$

the expected evolutionary trajectory of allele frequencies driven only by selection can be expressed as

(A1b) $$\zeta_{t}=\zeta_{0}-\sum_{i=0}^{t-1}s_{i},$$

with *p*_0_ being the frequency of the allele at time 0 (the first sampling point) and a positive value of *s_i_* implying that this allele is favored in interval *i*. In the following, it is assumed that there are *T* consecutive samples of the allele frequency, indexed as i=0,1,⋯,T−1, each based on a sample of *n* diploid individuals. Throughout, estimates of parameters are designated by a ^ symbol, with the estimate ($\hat{s}$) of the mean selection coefficient, *μ_s_*, being obtained by least-squares regression of the observed transformed variables ${\hat{\zeta }}_{t}$ on *t*.

The concern here is with the variance in the estimate $\hat{s}$ resulting from evolutionary variation in nature (which is a function of $\sigma_{s}^{2}$, the variance in *s* around its expected value, assumed here to be randomly distributed over time with independent values across sampling time points) and sampling variance (which is determined by the number of sample points, *T*, and their sample size, *n*). From Lynch and Walsh (1998; eq. A1.20a, with a correction for sampling bias that reduces the effective sample size from *T* to *T* – 1), the formula for the sampling variance of a regression coefficient leads to

(A2) $$\sigma^{2}(\hat{s})≃\frac{\sigma^{2}({\hat{\zeta }}_{t})[1-\rho^{2}({\hat{\zeta }}_{t},t)]}{(T-1)\sigma_{t}^{2}},$$

where $\sigma^{2}({\hat{\zeta }}_{t})$ is the variance in the observed values of ${\hat{\zeta }}_{t},\;\rho ({\hat{\zeta }}_{t},t)$ is their correlation with *t*, and assuming *T* consecutive samples,

(A3) $$\sigma_{t}^{2}=\frac{\left(T-1)(T+1\right)}{12}.$$

As the sample estimate ${\hat{\zeta }}_{t}$ is equal to the sum of the population parameter *ζ_t_* and a sampling deviation, its sampling variance has two components: 1) the evolutionary variance due to actual changes by selection and 2) the additional variance caused by finite numbers of individuals in the samples. The evolutionary variance is defined as

(A4) $$\sigma_{e}^{2}(\zeta )=E(\zeta^{2})-{\left[E(\zeta )\right]}^{2},$$

where *E* denotes an expectation. Using equation (A1b),

(A5) $$E(\zeta )=\frac{1}{T}\sum_{t=0}^{T-1}E(\zeta_{t})=\zeta_{0}-\frac{(T-1)\mu_{s}}{2}.$$

The expected squared value is

(A6a) $$E(\zeta^{2})=\frac{1}{T}\sum_{t=0}^{T-1}E(\zeta_{t}^{2})=\frac{1}{T}\sum_{t=0}^{T-1}{\left(\zeta_{0}-\sum_{i=0}^{t-1}s_{i}\right)}^{2},$$

and allowing for temporal variation in *s*, this reduces to

(A6b) $$E(\zeta^{2})=\zeta_{0}^{2}-\zeta_{0}\mu_{s}(T-1)+\frac{\mu_{s}^{2}(T-1)(2T-1)}{6}+\frac{\sigma_{s}^{2}(T-1)}{2}.$$

Substituting equations (A5 and A6b) into equation (A4),

(A7) $$\sigma_{e}^{2}(\zeta )=\left(\frac{T-1}{2}\right)\left[\frac{(T+1)\mu_{s}^{2}}{6}+\sigma_{s}^{2}\right].$$

Note that this derivation assumes that drift has a negligible effect on allele-frequency change during the time interval (0,T−1).

To obtain the additional variance in ${\hat{\zeta }}_{t}$ associated with finite sample size, we make use of the fact that for each estimate ${\hat{\zeta }}_{t}$,

(A8a) $$\sigma_{s}^{2}({\hat{\zeta }}_{t})≃\frac{1}{2np_{t}(1-p_{t})}$$

(Lynch 1987), where 2*n* is the number of alleles assayed in a sample of *n* diploid individuals, so that the average sampling variance over the time series is

(A8b) $$\sigma_{S}^{2}({\hat{\zeta }}_{t})≃\frac{1}{2Tn}\left[\sum_{t=0}^{T-1}\frac{1}{p_{t}(1-p_{t})}\right].$$

This expression can be further expanded by noting from equation (A1b) that

(A9) $$p_{t}=\frac{1}{1+f_{t}},$$

where $f_{t}=[(1-p_{0})/p_{0}]e^{-\mu_{s}t}.$ Using the fact that

(A10) $$\frac{1}{p_{0}(1-p_{0})}=\frac{1}{p_{0}}+\frac{1}{1-p_{0}}$$

then leads to

(A11a) $$\sigma_{S}^{2}({\hat{\zeta }}_{t})≃\frac{p_{0}}{2Tn(1-p_{0})}\sum_{t=0}^{T-1}e^{st}{\left(1+f_{t}\right)}^{2}.$$

For weak selection such that the allele frequency does not change dramatically over time interval *T*,

(A11b) $$\sigma_{S}^{2}({\hat{\zeta }}_{t})≃\frac{T-1}{2Tnp_{0}(1-p_{0})}.$$

The final component of equation (A2) requiring an expression involves the covariance between ${\hat{\zeta }}_{t}$ and *t*,

(A12) $$\sigma ({\hat{\zeta }}_{t},t)=E({\hat{\zeta }}_{t}\cdot t)-E({\hat{\zeta }}_{t})\cdot E(t).$$

Noting that

(A13) $$E({\hat{\zeta }}_{t}\cdot t)=\frac{\zeta_{0}(T-1)}{2}-\frac{\mu_{s}(T-1)(2T-1)}{6},$$

and using equation (A5) and E(t)=(T−1)/2 leads to

(A14) $$\sigma ({\hat{\zeta }}_{t},t)=-\frac{\mu_{s}(T-1)(T+1)}{12}.$$

One can see from equations (A3 and A14) that the slope of the regression $\sigma ({\hat{\zeta }}_{t},t)/\sigma_{t}^{2},$ has expected value $-\mu_{s}.$

Noting that equation (A2) expands to

(A15) $$\sigma^{2}(\hat{s})≃\left[\frac{1}{(T-1)\sigma_{t}^{2}}\right]\left[\sigma^{2}({\hat{\zeta }}_{t})-\frac{\sigma^{2}({\hat{\zeta }}_{t},t)}{\sigma_{t}^{2}}\right],$$

and substituting with the above expressions leads to equation (10) in the main text.

## References

[evaa056-B1] Bollback JP York TL Nielsen R. 2008 Estimation of 2*Nes* from temporal allele frequency data. Genetics 179(1):497–502. 18493066 10.1534/genetics.107.085019 PMC2390626

[evaa056-B2] Buffalo V Coop G. 2019 The linked selection signature of rapid adaptation in temporal genomic data. Genetics 213(3):1007–1045. 31558582 10.1534/genetics.119.302581 PMC6827383

[evaa056-B3] Charlesworth B. 2009 Fundamental concepts in genetics: effective population size and patterns of molecular evolution and variation. Nat Rev Genet. 10(3):195–205. 19204717 10.1038/nrg2526

[evaa056-B4] Crow JF Kimura M. 1970 An introduction to population genetics theory. New York: Harper and Row.

[evaa056-B5] Ferrer-Admetlla A Leuenberger C Jensen JD Wegmann D. 2016 An approximate Markov model for the Wright–Fisher diffusion and its application to time series data. Genetics 203(2):831–846. 27038112 10.1534/genetics.115.184598 PMC4896197

[evaa056-B6] Foll M Shim H Jensen JD. 2015 WFABC: a Wright–Fisher ABC-based approach for inferring effective population sizes and selection coefficients from time-sampled data. Mol Ecol Resour. 15(1):87–98. 24834845 10.1111/1755-0998.12280

[evaa056-B7] Hill WG. 1972 Estimation of realised heritabilities from selection experiments. I. Divergent selection. Biometrics 28(3):747–765. 5073250

[evaa056-B8] Hui TY Burt A. 2015 Estimating effective population size from temporally spaced samples with a novel, efficient maximum-likelihood algorithm. Genetics 200(1):285–293. 25747459 10.1534/genetics.115.174904 PMC4423369

[evaa056-B9] Karasov T Messer PW Petrov DA. 2010 Evidence that adaptation in *Drosophila* is not limited by mutation at single sites. PLoS Genet. 6(6):e1000924. 20585551 10.1371/journal.pgen.1000924 PMC2887467

[evaa056-B10] Kimura M. 1954 Process leading to quasi-fixation of genes in natural populations due to random fluctuation of selection intensities. Genetics 39(3):280–295. 17247483 10.1093/genetics/39.3.280 PMC1209652

[evaa056-B11] King EG Long AD. 2017 The Beavis effect in next-generation mapping panels in *Drosophila melanogaster*. G3 (Bethesda) 7:1643–1652. 28592647 10.1534/g3.117.041426 PMC5473746

[evaa056-B12] Krimbas CB Tsakas S. 1971 The genetics of *Dacus oleae.* V. Changes of esterase polymorphism in a natural population following insecticide control-selection or drift? Evolution 25(3):454–460. 28565021 10.1111/j.1558-5646.1971.tb01904.x

[evaa056-B13] Li H Durbin R. 2011 Inference of human population history from individual whole-genome sequences. Nature 475(7357):493–496. 21753753 10.1038/nature10231 PMC3154645

[evaa056-B14] Liu X Fu Y-X. 2015 Exploring population size changes using SNP frequency spectra. Nat Genet. 47(5):555–559. 25848749 10.1038/ng.3254 PMC4414822

[evaa056-B15] Long H, et al 2018 Evolutionary determinants of genome-wide nucleotide composition. Nat Ecol Evol. 2(2):237–240. 29292397 10.1038/s41559-017-0425-y PMC6855595

[evaa056-B16] Lynch M. 1987 Design and analysis of experiments on random drift and inbreeding. Genetics 120(3):791–807. 10.1093/genetics/120.3.791 PMC1203557 17246484

[evaa056-B17] Lynch M. 2007 The origins of genome architecture. Sunderland (MA): Sinauer Assocs., Inc.

[evaa056-B18] Lynch M Bost D Wilson S Maruki T Harrison S. 2014 Population-genetic inference from pooled-sequencing data. Genome Biol Evol. 6(5):1210–1218. 24787620 10.1093/gbe/evu085 PMC4040993

[evaa056-B19] Lynch M, et al 2016 Genetic drift, selection, and evolution of the mutation rate. Nat Rev Genet. 17(11):704–714. 27739533 10.1038/nrg.2016.104

[evaa056-B23] Lynch M, Walsh JB. 1998. Genetics and analysis of quantitative traits. Sunderland (MA): Sinauer Assocs., Inc.

[evaa056-B20] Malaspinas AS Malaspinas O Evans SN Slatkin M. 2012 Estimating allele age and selection coefficient from time-serial data. Genetics 192(2):599–607. 22851647 10.1534/genetics.112.140939 PMC3454883

[evaa056-B21] Maruki T Lynch M. 2015 Genotype-frequency estimation from high-throughput sequencing data. Genetics 201(2):473–486. 26224735 10.1534/genetics.115.179077 PMC4596663

[evaa056-B22] Nei M Tajima F. 1981 Genetic drift and estimation of effective population size. Genetics 98(3):625–640. 17249104 10.1093/genetics/98.3.625 PMC1214463

[evaa056-B24] Pearson K Jeffery GB Elderton EM. 1929 On the distribution of the first product moment-coefficient, in samples drawn from an indefinitely large normal population. Biometrika 21(1–4):164–201.

[evaa056-B25] Pollak E. 1983 A new method for estimating the effective population size from allele frequency changes. Genetics 104(3):531–548. 17246147 10.1093/genetics/104.3.531 PMC1202093

[evaa056-B26] Walsh JB Lynch M. 2018 Evolution and selection of quantitative traits. Oxford: Oxford University Press.

[evaa056-B27] Wang JL. 2001 A pseudo-likelihood method for estimating effective population size from temporally spaced samples. Genet Res. 78(3):243–257. 11865714 10.1017/s0016672301005286

[evaa056-B28] Waples RS. 1989 A generalized approach for estimating effective population size from temporal changes in allele frequency. Genetics 121(2):379–391. 2731727 10.1093/genetics/121.2.379 PMC1203625

[evaa056-B29] Wright S. 1948 On the roles of directed and random changes in gene frequency in the genetics of populations. Evolution 2(4):279–294. 18104586 10.1111/j.1558-5646.1948.tb02746.x

